# Exosomes in Age-Related Cognitive Decline: Mechanistic Insights and Improving Outcomes

**DOI:** 10.3389/fnagi.2022.834775

**Published:** 2022-03-01

**Authors:** Michael R. Duggan, Anne Lu, Thomas C. Foster, Mathieu Wimmer, Vinay Parikh

**Affiliations:** ^1^Department of Psychology and Neuroscience Program, Temple University, Philadelphia, PA, United States; ^2^Department of Neuroscience, University of Florida College of Medicine, Gainesville, FL, United States

**Keywords:** exosomes, aging, cognition, biomarkers, therapeutics

## Abstract

Aging is the most prominent risk factor for cognitive decline, yet behavioral symptomology and underlying neurobiology can vary between individuals. Certain individuals exhibit significant age-related cognitive impairments, while others maintain intact cognitive functioning with only minimal decline. Recent developments in genomic, proteomic, and functional imaging approaches have provided insights into the molecular and cellular substrates of cognitive decline in age-related neuropathologies. Despite the emergence of novel tools, accurately and reliably predicting longitudinal cognitive trajectories and improving functional outcomes for the elderly remains a major challenge. One promising approach has been the use of exosomes, a subgroup of extracellular vesicles that regulate intercellular communication and are easily accessible compared to other approaches. In the current review, we highlight recent findings which illustrate how the analysis of exosomes can improve our understanding of the underlying neurobiological mechanisms that contribute to cognitive variation in aging. Specifically, we focus on exosome-mediated regulation of miRNAs, neuroinflammation, and aggregate-prone proteins. In addition, we discuss how exosomes might be used to enhance individual patient outcomes by serving as reliable biomarkers of cognitive decline and as nanocarriers to deliver therapeutic agents to the brain in neurodegenerative conditions.

## Introduction

Age-related cognitive decline remains a prominent public health concern, with Alzheimer’s Disease (AD) accounting for the majority of dementia diagnoses (i.e., 60–80%) (Alzheimer’s Association, 2020). Despite the recent approval of an anti-amyloid biologic (i.e., aducanumab), it poses significant risks for side effects (e.g., microhemorrhages, edema), can be cost prohibitive due to the lack of insurance coverage and has not yet been shown to reliably mitigate cognitive decline (Alexander et al., 2021; Anderson et al., 2021; Knopman et al., 2021; Salloway et al., 2022). As alternative approved pharmacotherapies (i.e., AChE inhibitors and an NMDAR antagonist) can only ameliorate symptomology temporarily, there remains no treatment capable of preventing or curing AD and its associated cognitive decline (Bullain and Doody, 2020).

Aging is the most prominent risk factor for cognitive decline, but behavioral symptomology and underlying neurobiology can vary between individuals. Indeed, some individuals exhibit significant cognitive impairments in comparison to age-matched controls, while others maintain intact cognitive functioning or display only minimal decline (Wilson et al., 2002; Hayden et al., 2011). Although elderly individuals with poor cognitive performance are at a higher risk of developing age-related dementia including AD (Albert et al., 2001), examinations from comprehensive clinical samples indicate such age-related cognitive variation can occur independently from pathological manifestations (i.e., cognitive deficits without neuropathological hallmarks and vice versa) (Katzman et al., 1988; Morris et al., 1996; Balasubramanian et al., 2012; Negash et al., 2013; Kawas et al., 2015; Aiello Bowles et al., 2019). Furthermore, after accounting for known pathological measures, approximately half of the estimated variation in cognitive decline remains unexplained (Boyle et al., 2013, 2021). In fact, postmortem analyses now suggest the behavioral symptomology leading to dementia diagnosis is often accompanied by a mixture of neuropathological hallmarks (Schneider et al., 2007; Rahimi and Kovacs, 2014; James et al., 2016; White et al., 2016). While these results exemplify the need for a better understanding of the biological mechanisms that contribute to differing cognitive trajectories in aging, they also suggest opportunities to improve diagnostic indicators for cognitive decline associated with age-related neurodegenerative disorders.

Several approaches have proved valuable for improving diagnostics and developing novel therapeutics. For one, the implementation of novel PET ligands over the past decade for Aβ and Tau is now recognized as an important component in recently adopted AD criteria (Jack et al., 2016). However, the detection of abnormal protein aggregates via PET has several limitations, including its predictability with regard to behavioral symptomology over time (Jack et al., 2019). Furthermore, these methods are primarily utilized for screening participants or assessing endpoints in clinical trials, in part due to prohibitive costs not covered by insurance as well as a lack of standardized thresholds to differentiate between dementia stages (Mitka, 2013; Rice and Bisdas, 2017; Weigand et al., 2020). Along with PET imaging, genomic interrogations of large clinical cohorts have further substantiated the increased risk for cognitive decline among individuals with common (e.g., *APOE*, *APP*, *PSEN1* etc.) as well as rare (e.g., *TREM2*, *MS4A*, *SORL1* etc.) genetic variants (Lanoiselée et al., 2017; Deming et al., 2019; Yamazaki et al., 2019; Holstege et al., 2020). However, the application of these findings to improve patient outcomes requires further elucidation (Van Cauwenberghe et al., 2016; Patron et al., 2019; Novikova et al., 2021). In addition, the advent of advanced proteomic platforms has improved diagnostic capacity compared to clinical assessment, particularly for CSF and blood derived samples; however, these can sometimes rely on a limited set of molecular targets measured from a single time point and may not necessarily relay information regarding the causal, biological mechanisms that facilitate variation in cognitive functioning (Janelidze et al., 2020; Palmqvist et al., 2020; Thijssen et al., 2020). While these approaches can help identify individuals at risk for cognitive deficits in aging, one approach that is increasingly recognized to gain insights into the neurobiological mechanisms of cognitive decline while potentially improving individual outcomes is the analysis of extracellular vesicles, namely exosomes.

In the current review, we will highlight recent findings which illustrate how the analysis of exosomes can improve our knowledge of varying mechanisms underlying age-related cognitive decline. In addition, we will discuss how exosomes might be used to enhance individual patient outcomes by serving as reliable biomarkers and therapeutic agents for age-related neurodegenerative disorders.

## Exosomes

Exosomes are membranous lipid compartments (∼50–150 nm) whose primary function is to translocate biological substrates (e.g., proteins, lipids, nucleic acids) between cells (Tkach and Thery, 2016; van Niel et al., 2018). Exosome biogenesis begins at the plasma membrane with the formation of early-sorting endosomes (ESEs). ESEs are formed when plasma membrane invagination encloses cell-surface proteins and other proximal molecules in the extracellular environment. These may combine with existing ESEs, while the Golgi and ER can also contribute to their contents (Hessvik and Llorente, 2018; van Niel et al., 2018; Mathieu et al., 2019). ESEs can eventually develop into late-sorting endosomes (LSEs), whose further inward invagination generates multivesicular bodies (MVBs) that contain intraluminal vesicles (ILVs). From here, MVBs can either fuse with lysosomes or autophagosomes for degradation, or fuse with the plasma membrane for extracellular release of ILVs, which become *de facto* exosomes upon exiting the cell (Gurunathan et al., 2019). While the exact processes governing their intercellular transport remain debated, exosomes can be internalized through several distinct mechanisms, including clathrin-dependent endocytosis and phagocytosis, which results in the release of exosomal contents in recipient cells (Mulcahy et al., 2014). Of note, the current article will primarily focus on exosomes, rather than on extracellular vesicles (EVs), which is a general term used to describe different subtypes of vesicles based on their size, content, and function, including exosomes, microvesicles (MVEs), and apoptotic bodies. For example, although exosomes are routinely classified alongside MVEs, their biogenesis is thought to utilize distinct cellular mechanisms (i.e., outward budding and fission of the plasma membrane) (Tricarico et al., 2017). Regarding nomenclature, the current review will utilize the terminology of exosome throughout, since the literature cited herein uses the term exosome or small EV when referring to the same type of vesicle; however, guidelines suggest that the later term is the appropriate generic term (Théry et al., 2018).

Exosome production, release and uptake is thought be conserved across cell types, including cells of the central nervous system (CNS) (i.e., neurons, astrocytes, oligodendrocytes, microglia etc.) (Potolicchio et al., 2005; Fauré et al., 2006; Krämer-Albers et al., 2007; Goetzl et al., 2018b). Renewed interest in this class of extracellular vesicles, coupled with technological advances, has resulted in a variety of biochemical techniques to facilitate exosome analysis, including high-sensitivity commercially available assays at a relatively low cost (Patel et al., 2019). Depending on the inherent properties of the sample (e.g., *in vitro* supernatant *vs*. clinical plasma/serum), processing generally involves isolation (e.g., ultracentrifugation, size exclusion chromatography, immunoaffinity, polymer precipitation etc.) and characterization (e.g., nanoparticle tracking, resistive pulse sensing, atomic force microscopy etc.) followed by biological interpretation of exosomal quantity and contents utilizing complementary biochemical techniques (e.g., high-throughput sequencing, proteomic arrays etc.) (Gurunathan et al., 2019). Given that differing cell types incorporate varying molecular components during endosomal sorting and exosome release, investigators can utilize this heterogeneity as molecular signatures to determine their cellular origin. Thus, exosomes released by neurons, astrocytes, oligodendrocytes, microglia, endothelial cells and pericytes can be differentiated according to antibody-based purification utilizing L1CAM (L1 cell adhesion molecule), GLAST (Glutamate aspartate transporter), PLP1 (Proteolipid protein 1), TMEM119 (Transmembrane protein 119), CD31 (cluster of differentiation 31) and PDGFRβ (platelet-derived growth factor receptor beta), respectively (Frühbeis et al., 2020; Kumar et al., 2020). The capacity to identify a given exosome’s parent cell is particularly valuable given that peripheral exosomes can penetrate the blood-brain-barrier (BBB), while brain derived exosomes can be readily detected in systemic samples (Alvarez-Erviti et al., 2011; Banks et al., 2020; Kumar et al., 2021). It should be noted that a recent investigation suggested L1CAM may not be an appropriate marker to identify neuronal specific exosomes (Norman et al., 2021); however, the interpretation of such data as well as the reliability of these findings remain contested, including contrary evidence of L1CAM positive vesicles enriched for neuronal proteins (e.g., BDNF, neuronal enolase) (Mustapic et al., 2017; Suire et al., 2017).

Attempts have been made to categorize exosomes according to their net advantages or disadvantages for CNS homeostasis, yet such a dichotomous distinction may be misleading. For instance, accumulating evidence illustrates exosomes facilitate symbiotic dynamism between neurons and oligodendrocytes, whereby activity dependent synaptic transmission triggers exosomal secretion from oligodendrocytes that contain essential myelin components (e.g., myelin basic protein [MBP], myelin oligodendrocyte glycoprotein [MOG] etc.); subsequently, internalization of these exosomes amongst proximal neurons enhances cellular viability (Domingues et al., 2020). Conversely, data suggest exosomes can serve as pathological conduits, capable of spreading aggregate-prone polypeptides between cells in a prion-like manner, including Aβ oligomers and pTau (Sardar Sinha et al., 2018; Polanco et al., 2021). However, exosome biology is itself an evolutionary conserved process intertwined with a myriad of other fundamental cellular mechanisms, ranging from gene expression to lysosomal autophagy (Kalluri and LeBleu, 2020). Therefore, these extracellular vesicles could presumably represent causal pathogenic processes leading to cognitive decline, compensatory responses to confer neuroprotection or a common outcome from upstream cellular mechanisms (i.e., epiphenomenon). As exosomes likely serve varying functions under differing physiological conditions, the following sections defer from categorizing exosomes according to their net advantages or disadvantages for CNS homeostasis and instead highlight recent advances in our understanding for the role of exosomes in age-related cognitive decline.

## Insights for Neurobiological Mechanisms of Cognitive Decline

### MicroRNAs

A large quantity of exosome research has focused on their capacity to incorporate and transport microRNAs (miRNAs). These short (18–25 base pairs in length; 22 avg.) non-coding RNAs function as post-transcriptional regulators of gene expression by binding to complementary pairing sites on mRNA (e.g., 3′ UTR, 5′ UTR, coding sequences etc.) across various locations within a cell, including the nucleus, cytoplasm and subcellular compartments, such as stress granules (O’Brien et al., 2018). Such binding subsequently influences expression of the targeted mRNAs, most notably by triggering their degradation and silencing the expression of transcripts which would otherwise be translated (Huntzinger and Izaurralde, 2011; Ipsaro and Joshua-Tor, 2015). Through these mechanisms, miRNAs maintain the capacity to influence a plethora of downstream processes that are dependent on gene expression, ranging from energy utilization to cellular growth. For example, miR-193b binds to the 3′ UTR of Amyloid Precursor Protein (APP) mRNA and represses its ensuing protein expression; interestingly, this miRNA also maintains significantly lower exosomal concentrations among AD patients, suggesting its low levels may exacerbate the generation of Aβ (Liu et al., 2014; Yang et al., 2018). Although additional studies are required to fully elucidate the functional implications of miRNAs, their ubiquitous expression across varying physiological conditions coupled with their incorporation into exosomes has made them an attractive target for examining the underlying mechanisms of age-related cognitive decline.

Several studies have characterized systemic exosomal miRNAs among elderly individuals to identify potential downstream biological pathways that might facilitate late life cognitive dysfunction. Utilizing plasma-derived samples enriched with exosomes from healthy aged participants, one recent study analyzed miRNAs via multiplex sequencing and correlated their levels with performance on the Montreal Cognitive Assessment (MoCA). While a large set of miRNAs showed significant positive correlations with age, another set maintained significant negative correlations with cognitive performance, including those miRNAs selectively expressed in the brain. In turn, functional annotation of miRNAs associated with poor performance revealed several biological pathways, including neurotrophin signaling, whose miRNA-mediated regulation may be important mediators of cognitive decline (Rani et al., 2017). Furthermore, machine learning approaches indicated expression levels of such miRNAs could predict several other cognitive outcomes, including fluid, crystallized and overall cognition (Gullett et al., 2020). Reanalysis of the same data set using weighted gene co-expression network analyses (WGCNA) coupled with functional enrichment revealed modules of miRNAs whose regulation of cancer-related signaling pathways may also account for MoCA performance among elderly participants (Ye et al., 2020). Such results complement another study which compared exosome enriched CSF samples from cognitively intact elderly individuals to those diagnosed with AD. Here, AD individuals exhibited elevated exosome concentrations of miRNAs previously implicated in cognitive decline (i.e., miR-9-5p and miR-598). Given that complementary *in silico* analysis linked these miRNAs to stress response and neurotrophic signaling pathways, findings suggested miRNA mediated regulation of these biological processes may increase risk for cognitive decline (Riancho et al., 2017). Interestingly, exosomal miRNA regulation of such specific pathways (i.e., neurotrophic signaling, cancer-related signaling and stress response) has been reported in other investigations, suggesting these processes might constitute common biological mechanisms that are associated with late life cognitive dysfunction (Gui et al., 2015; McKeever et al., 2018; Gámez-Valero et al., 2019; Dong et al., 2021).

To gain more reliable insights into the neurobiological mechanisms of cognitive decline, additional investigations have interrogated the role of miRNAs from brain derived exosomes. After isolating neural exosomes from AD plasma samples, researchers recently identified a set of significantly upregulated (i.e., miR-23a-3p, miR-223-3p, and miR-190a-5p) and downregulated (i.e., miR-100-3p) miRNAs which were predicted to regulate several homeostasis pathways in the CNS, including steroid biosynthesis and mTOR signaling (Serpente et al., 2020). In another recent investigation, neural exosomes isolated from plasma samples revealed two miRNAs (i.e., miR-132 and miR-212) whose degree of downregulation was capable of distinguishing AD stages (i.e., AD vs. MCI vs. Control). Although investigators here inferred the biological relevance of these miRNAs, potential regulation of specific downstream processes was not examined (Cha et al., 2019).

Preclinical investigations have also characterized exosomal miRNAs to identify potential biological pathways that might facilitate cognitive decline. Utilizing exosomes harvested from hippocampal stem cell cultures, a group of researchers identified a set of miRNAs (i.e., miR-17, miR-322, and miR-485) whose *in vivo* delivery via exosomes was capable of ameliorating memory deficits in rodents (i.e., Novel Object Recognition) otherwise induced by intracerebral application of Aβ oligomers; furthermore, researchers inferred these benefits may be attributed to the increased synaptic phosphorylation of calmodulin-dependent kinase II (Micci et al., 2019). Similar results have been noted among *in vitro* studies, whereby the transfer of miRNAs via exosomes from healthy stem cells can induce significantly improved viability in recipient neural cells by modulating alternative intracellular signaling cascades (e.g., PTEN/PI3K/Akt pathway) (Wei et al., 2020; Cong et al., 2021).

To expand our understanding for the role of exosomal miRNAs in mediating the underlying mechanisms of cognitive decline, several lines of inquiry require elucidation, some of which are addressed below. For one, we currently lack a comprehensive understanding of the mechanisms within recipient cells that are modulated by exosomal miRNAs. Previous evidence suggests miRNAs released by exosomes remain functional in their recipient cells, capable of directly downregulating the expression of target transcripts, similar to endogenous effects in parent cells (Umezu et al., 2013; Zhou et al., 2014). Interestingly, exosomal miRNA can also influence mechanisms in recipient cells other than gene expression, such as serving as ligands for Toll-Like Receptors in intracellular endosomes and triggering their activation (Fabbri et al., 2012). Given that prior studies have disproportionately relied on cellular models of peripheral origin, it would be beneficial to discern the precise cellular mechanisms altered by exosomal miRNAs as well as assess if such effects differ depending on CNS cell type. Related to this gap in knowledge, observations indicate the ratio of a given miRNA copy to the number of exosomes is substantially below one, with an average of one per 121 exosomes (Chevillet et al., 2014). This suggests additional investigations should more accurately quantify the association between exosomal miRNA content and ensuing functional alterations in CNS cells, such as effects of a single miRNA from one exosome and synergistic effects of multiple miRNAs from different exosomes. Along with miRNAs, accumulating data indicate exosomes can translocate other types of non-coding RNAs, including long non-coding RNAs (lncRNAs), ribosomal RNAs and circular RNAs (Huang et al., 2013; Li et al., 2015). By examining how these other exosomal non-coding RNAs interact with miRNAs to exert variation in CNS processes, investigators will likely gain a greater understanding of the neurobiological mechanisms contributing to cognitive decline in late life.

### Neuroinflammation

A mild state of chronic neuroinflammation in the absence of overt infection referred to as sterile inflammation, or *inflamm-aging*, is a common feature of normal aging. Higher levels of neuroinflammation are a risk factor for age-related cognitive decline and the accumulation of neuropathological hallmarks (Wyss-Coray and Rogers, 2012; Franceschi and Campisi, 2014; Ransohoff, 2016). Neuroinflammation can be quantified through a diverse set of variables, including levels of inflammatory messengers (e.g., cytokine, chemokines, prostaglandins) and phenotypic characteristics of CNS cells which mediate immune processes (e.g., microglia, astrocytes) (Glass et al., 2010). In the event of infection or injury, optimal neuroinflammation can promote neuroprotective effects, yet its dysregulation from a variety of factors in aging (e.g., increased iron load, accumulation of lipid droplets, oxidative stress etc.) can result in an aberrant, pro-inflammatory state (Wong, 2013; Ransohoff, 2016; Rea et al., 2018). In turn, this can induce deleterious consequences for homeostatic processes in CNS cells and contribute to an increased risk for cognitive decline and neurodegeneration. Investigations utilizing a variety of techniques (e.g., genomic, neuroimaging etc.) across experimental designs (i.e., clinical and preclinical) support the association between increased neuroinflammation and late life cognitive dysfunction (He et al., 2007; Cribbs et al., 2012; Kreisl et al., 2013; Elmore et al., 2018; Duggan and Parikh, 2021).

The modulation of neuroinflammatory processes by exosomes has been increasingly scrutinized in the context of several conditions, including neurodegenerative diseases and traumatic brain injury (Gupta and Pulliam, 2014; Thomi et al., 2019; Kumar et al., 2021). A recent interrogation elegantly addressed this mechanism by isolating astrocyte and neuronal exosomes from plasma samples of AD patients and examining their subsequent *in vitro* effects on several neuronal populations, including rat cortical neurons as well as human iPSC-derived neurons. Compared to age-matched and cognitively sound participants, brain derived exosomes from AD individuals triggered a robust activation of the complement cascade (i.e., an immune response pathway for host defense against infections), which ultimately resulted in the disruption of plasma membrane integrity as well as elevated neurotoxicity (Nogueras-Ortiz et al., 2020). Such findings were consistent with results from another study, which illustrated increased concentrations of multiple complement cascade proteins in astrocyte derived exosomes from AD individuals, including C1q and C4b (Goetzl et al., 2018b).

The role of exosomes in mediating neuroinflammatory mechanisms underlying cognitive decline has disproportionally relied on preclinical models, particularly transgenic animals. In APP/PS1 double transgenic mice, recent investigations have shown the administration of exosomes from healthy, bone marrow derived mesenchymal stem cells can normalize neuroinflammation across the cortex and hippocampus [i.e., decrease pro-inflammatory cytokines (TNF-α, IL-1β, and IL-6); increase anti-inflammatory cytokines (IL-10, IL-4, and IL-13)]; in turn, this is associated with the mitigation of pathological peptides (i.e., Aβ concentrations, plaque deposition) and the preservation memory capacities (i.e., Morris Water Maze). Interestingly, such beneficial effects on cognition and neuroinflammation were comparable when exosomes were specifically targeted to the brain or administered systemically, highlighting the capacity of these EVs to exert their effects across the BBB (Cui et al., 2018, 2019). Follow-up studies have expounded on these findings by illustrating the exosomal moderation of neuroinflammatory processes and ensuing benefits to cognitive performance may be attributed to effects on microglia specifically (i.e., the resident macrophage of the CNS) (Ding et al., 2018; Li et al., 2020; Zhang et al., 2021).

To enhance our understanding of exosomal-mediated neuroinflammation and associated risk for cognitive decline, several lines of evidence require further interrogation. Most prominently, further studies are needed to characterize the contents of exosomes which are responsible for alterations in CNS inflammation. Along with pathogens (e.g., bacteria, viruses), a variety of molecules can induce immune responses in the brain, including reactive oxygen species (ROS), purine metabolites (e.g., urate crystals), calcium-binding proteins and lipotoxic ceramides (Ransohoff and Perry, 2009; Chen and Nunez, 2010; Goldberg and Dixit, 2015). In addition to screening for exosomal contents, investigations should distinguish how these molecules delivered to the intracellular environment differentially trigger inflammatory responses compared to their previously characterized effects, many of which have been assessed at the extracellular surface (e.g., cytokines binding to receptors). Evidence also indicates inflammation emanating from the periphery can increase the risk for late life cognitive impairments, either by lowering the threshold for activation in the CNS or exacerbating ongoing neuroinflammation (Holmes et al., 2009; Cunningham, 2013; Widmann and Heneka, 2014; Barter et al., 2021). Indeed, data suggest exosomes can be integral to this process: epithelial cells at the choroid plexus increase production of exosomes containing proinflammatory miRNAs in response to increased systemic inflammation (Balusu et al., 2016). Given that exosomes themselves can also exert their effects across the BBB, further studies should assess how peripheral inflammatory states facilitate variation in neuroinflammation via exosomes and increase the likelihood for cognitive deterioration in elderly individuals.

### Aggregate-Prone Proteins

Evidence indicating the association between aggregate-prone proteins (i.e., Aβ, pTau) and age-related cognitive decline is abundant, with increasing data suggesting their spread between CNS cells can be facilitated through exosomes (Wang et al., 2017; Sardar Sinha et al., 2018). It should be reiterated that cognitive decline can occur independent from measures of neuropathology, while behavioral symptomology is often accompanied by a mixture of neuropathological hallmarks (White et al., 2016; Aiello Bowles et al., 2019). Although debate persists regarding whether these polypeptides are pathogenic, neuroprotective or epiphenomena of upstream mechanisms, it is thought their abnormal abundance and ensuing aggregation (i.e., due to increased production and/or decreased degradation) can perturb homeostatic processes in CNS cells, facilitate neuronal malfunctioning and contribute to the manifestation of behavioral symptomology (Espay et al., 2019; Mathieu et al., 2020). Rather than discuss the causal relevance of these proteins, the subsequent section instead focuses on evidence of their transport within exosomes and implications for the biological underpinnings of cognitive decline.

Several investigations have leveraged aggregate-prone proteins in exosomes to predict individual variation in cognitive decline as well as their capacity to mediate its underlying neurobiological mechanisms. In one study with a 3 year follow up period, levels of Aβ_1–42_, pTau-181 and pTau-S396 in neuronal exosomes isolated from blood samples could differentiate AD patients from controls, as well as those patients with MCI who remained cognitively stable from those who illustrated progressively declining MMSE scores. Furthermore, intracerebral injection of such exosomes into healthy adult mice induced a significant accumulation of pTau in CA1 pyramidal neurons, compared to those exosomes from stable MCI participants (Winston et al., 2016). For Aβ_1–42_ specifically, data indicate its levels in neuronal exosomes offers similar capacity to predict deterioration in cognitive performance; among participants diagnosed with MCI according to a battery of tests (e.g., MMSE, MoCA, ADAS-cog, AVLT), individuals who illustrated higher exosomal Aβ_1–42_ levels maintain an 11.1- and 8.5-fold increased risk for significant cognitive deterioration at 2 and 3 year follow-up assessments, respectively, compared to MCI participants who remained cognitively stable (Zhao et al., 2020). Similar capacity to predict longitudinal cognitive performance has been noted for neuronal exosome concentrations of total Tau and pTau (Nam et al., 2020). Furthermore, *in vitro* and *in vivo* investigations have demonstrated Aβ as well as Tau in brain derived exosomes can readily compromise neuronal viability and induce cell death (Winston et al., 2019; Elsherbini et al., 2020a,b; Ruan et al., 2020). Together, these data suggest exosomal concentrations of aggregate-prone peptides can induce aberrant biological consequences in CNS cells (e.g., protein aggregation, apoptosis etc.) and may constitute an important mechanism leading to increased risk for cognitive impairments in aging.

Although exosomal transfer of aggregate-prone peptides may be an important mechanism underlying cognitive decline, several related processes require further investigation. Notably, data have yet to elucidate how exosomal propagation of other aggregate-prone peptides (i.e., TDP-43, α-synuclein) may synergistically compromise CNS homeostasis (Iguchi et al., 2016; Ngolab et al., 2017; Niu et al., 2020; Zhang et al., 2020). Such a gap in knowledge is particularly relevant provided that age-related cognitive deficits are often associated with the accumulation of multiple species of aggregate-prone peptides (Schneider et al., 2007; Rahimi and Kovacs, 2014; James et al., 2016; White et al., 2016). Additional experiments should also examine how the uptake of pathologically relevant proteins by microglia differentially contributes to the clearance of such peptides, as well as their potential spread to proximal cells via exosomes. As microglia inherently play a role in Aβ/Tau degradation via phagocytosis, and can facilitate the spread of aggregate-prone peptides via the secretion of Aβ/Tau-containing EVs, it remains unknown to what degree microglia contribute both to the degradation of such proteins as well as their propagation via exosomes (Joshi et al., 2014; Asai et al., 2015; Gouwens et al., 2018). In addition, studies should determine if the transfer of transcripts encoding for aggregate-prone peptides (e.g., *APP*, *MAPT*) via exosomes is capable of increasing translation of these proteins in recipient cells; such exploitation of host cell gene expression machinery could facilitate the accumulation of protein aggregates in otherwise healthy CNS cells (Gui et al., 2015; Khan et al., 2016; O’Brien et al., 2020). Another unique aspect that requires elucidation is the attachment of aggregate-prone proteins to the surface of exosomes, rather than their inclusion into the exosomal milieu. For instance, evidence suggests Aβ as well as its degradation enzymes can be attached to the membrane surface of exosomes (Bulloj et al., 2010; Yuyama et al., 2014; Lim et al., 2019). Further investigations are encouraged to determine how aggregate-prone proteins, attached to the surface of exosomes, confer effects on the extracellular environment (e.g., extra-cellular matrix dysregulation, plaque deposition etc.) and whether these effects increase the likelihood for cognitive deterioration in aging.

## Using Exosomes to Improve Outcomes in Cognitve Decline

### Exosomes as Biomarkers to Predict Dementia

Predicting individual cognitive trajectories in aging can be important for diagnostic purposes, and the adoption of exosomes as biomarkers may prove particularly useful in this respect. Clinical assessment alone can fail to discriminate those individuals who maintain cognitive resilience from those who develop MCI, as well as those MCI patients who go on to develop dementia (Frölich et al., 2017; Sabbagh et al., 2017; Albert et al., 2018). Although PET neuroimaging and CSF assessments via lumbar puncture can augment diagnostic sensitivity and specificity of tests to detect plaques and tangles, such approaches can be invasive, lengthy and costs are not routinely covered by insurance. Furthermore, early detection of vulnerable individuals may help improve patient outcomes and offer an opportunity for earlier interventions. Indeed, the only FDA approved treatments for dementia (i.e., cholinesterase inhibitors, NMDA antagonist, Aβ antibodies) are specified for persons already exhibiting symptoms; however, the underlying neurobiology of cognitive decline is hypothesized to begin years or decades before the onset of behavioral impairments (Long and Holtzman, 2019). Therefore, using exosomes as biomarkers to enhance detection of susceptible individuals may facilitate intervention by health care providers (e.g., enrolling in clinical trials, implementing risk-reduction strategies) and ultimately improve prognosis (Ngandu et al., 2015; Seifan and Isaacson, 2015).

Numerous studies have used miRNAs isolated from blood-derived exosomes as biomarkers to classify individuals who are exhibiting late life cognitive dysfunction. Table 1 summarizes clinical investigations that have developed algorithms based on machine learning and other statistical approaches to predict diagnostic status using miRNA from exosomes. Altered expression of larger (i.e., 7–16) as well as more limited (i.e., 1–3) panels of specific miRNAs are capable of accurately distinguishing between clinical cases with AD dementia and healthy, aged-matched controls (Liu et al., 2014; Cheng et al., 2015; Lugli et al., 2015; Wei et al., 2018; Dong et al., 2021). In addition, miRNAs from blood-derived exosomes have also been used to differentiate dementia phenotypes (i.e., AD dementia, Lewy Body dementia, Vascular dementia etc.) (Yang et al., 2018; Gámez-Valero et al., 2019; Barbagallo et al., 2020). Although several studies have directly associated exosomal miRNAs with acute cognitive performance (e.g., MoCA, MMSE, CDR), further evidence is needed to extrapolate such associations with performance over time (Rani et al., 2017; Wei et al., 2018; Gullett et al., 2020).

**TABLE 1:** Clinical studies using exosome-derived microRNAs to predict dementia.

Source of exosome	microRNA (miR)	Prediction model/diagnostic evaluation	Biomarker accuracy	Clinical classification	References
Serum	**Upregulated in AD:** miR18b-5p, miR-20a-5p, miR30e-5p, miR-582-5p, miR-106a-5p, miR-361-5p, miR-143-3p, miR-424-5p, miR-93-5p, miR-106b-5p, miR-101-3p, miR-335-5p, miR-15a-5p **Downregulated in AD:** miR-1306-5p, miR342-3p, miR-15b-3p	Random decision forest	Combined panel of 16 miRs: sensitivity 87%;specificity 77%	HC vs. AD	Cheng et al., 2015
Serum	**Upregulated in AD:** miR-22-3p, miR-378a-3p **Downregulated in AD:** miR-30b-5p	Logistic regression, ROC curve analysis	Combined panel of 3 miRs: AUC 0.88	HC vs. AD	Dong et al., 2021
Serum	**Downregulated in AD:** miR-223	Spearman correlation, ROC curve analysis	MMSE scores (*r* = 0.37); CDR scores (*r* = 0.46); AUC 0.88	HC vs. AD	Wei et al., 2018
Serum	**Upregulated in AD:** miR-135a, miR-384 **Downregulated in AD:** miR-193b	ROC curve analysis	miR-135a (AUC 0.72), miR-193b (AUC 0.55), miR-384 (AUC 0.99)	AD vs. VD	Yang et al., 2018
Serum	**Upregulated in AD:** miR-22, miR-23a, miR-29a, miR-34b, miR-130b	Logistic regression, ROC analysis	Univariate logistic regression and ROC curve for each miR: miR-22 (AUC 0.76), miR-23a (AUC 0.82), miR-29a (AUC 0.83), miR-34b (AUC 0.81), miR-130b (AUC 0.80) Combined panel of 5 miRs: AUC 0.85	AD vs. VD	Barbagallo et al., 2020
Serum & Plasma	**Downregulated in AD:** miR-193b	Mann-Whitney U Test	miR-193b (*p* < 0.05)	HC vs. AD HC vs. MCI MCI vs. AD	Liu et al., 2014
Plasma	**Downregulated in AD:** miR-342-3p, miR-141-3p, miR-342-5p, miR-23b-3p, miR-24-3p, miR-125b-5p, miR-152-3p	J48 decision tree, SVM, adaboostM1, ROC curve analysis	Combined panel of 7 miRs: precision (J48 decision tree 0.78, SVM 0.82, AdaboostM1 0.89); AUC (J48 decision tree 0.75, SVM 0.83, AdaboostM1 0.92)	HC vs. AD	Lugli et al., 2015
Plasma	**Downregulated in AD:** miR-451a, miR-21-5p	ROC curve analysis	miR-451a (AUC 0.95), miR-21-5p (AUC 0.93)	AD vs. DLB	Gámez-Valero et al., 2019
Plasma	**Upregulated miRs correlated with lower MoCA scores:** miR-342-3p, miR-125b-5p, miR-10a-5p, miR-140-3p, miR-451a, miR-99a-5p, miR-23b-3p, miR-10b-5p, miR-125a-5p, miR-186-5p, miR-378a-3p, miR-26b-5p, miR-30c-5p	Multiple regression	Coefficients (age –0.063 to –0.074; miR –1.00 to –1.74); R^2^ 0.12 to 0.15; *p* < 0.05 to 0.01	Older adults (60–89 years)	Rani et al., 2017
CSF	**Downregulated in AD:** miR-29c, miR-136-3p, miR-16-2, miR-331-5p **Upregulated in AD:** miR-132-5p, miR-485-5p	ANOVA	Fold Change miR-29c (–0.47), miR-136-3p (–0.12), miR-16-2 (–0.83), miR-331-5p (–0.61), miR-132-5p (0.12), miR-485-5p (1.39)	HC vs. AD	Gui et al., 2015
CSF	**Downregulated in AD:** miR-451a, miR-605-5p **Upregulated in AD:** miR-125b-5p	ROC curve analyses	AUC (EOAD/LOAD) miR-451a (0.95/0.85) miR-605-5p (0.71/0.77) miR-125b-5p (0.72/0.79)	HC vs. LOAD HC vs. YOAD	McKeever et al., 2018
CSF	**Downregulated in AD:** miR-193b	Spearman correlation	CSF Aβ load (*r* = –0.44, *p* < 0.05)	AD	Liu et al., 2014

*AD, Alzheimer’s Disease; AUC, area under the curve; CDR, clinical dementia rating; CSF, cerebrospinal fluid; DLB, dementia with Lewy bodies; HC, Healthy Controls; LOAD, Late-Onset AD; MCI, mild cognitive impairment; MMSE, Mini-Mental State Examination; MoCA, Montreal Cognitive Assessment; ROC, receiver operating characteristic; SVM, support vector machine, VD, vascular dementia; YOAD, Young-Onset AD.*

*The direction of change in microRNA is indicated in bold.*

Compared to blood samples, available evidence suggests measures of exosomal miRNAs obtained from CSF samples offer similar accuracies in distinguishing individuals with age-related cognitive impairment (Liu et al., 2014; Gui et al., 2015; McKeever et al., 2018). However, results from several studies highlight important considerations for miRNA biomarker assessment in CSF exosomes. For example, while lower levels of miR-193b in exosomes derived from serum, plasma or CSF can differentiate AD patients from controls, levels of miR-193b in CSF exosomes can discriminate AD patients more accurately than levels from serum exosomes (i.e., 71.4% vs. 58.8%) (Liu et al., 2014). In addition, when miRNA measurements are derived from CSF, it should be noted that differential concentrations of some miRNAs in AD subjects may illustrate opposite patterns of expression depending on whether such miRNAs are encapsulated in exosomes or soluble in CSF (Riancho et al., 2017). Regarding non-miRNA biomarkers, measurements of AD-specific biomarkers (i.e., Aβ, pTau) maintain robust correlations across blood and CSF samples, thereby offering similar capacities to discriminate participants with age-related cognitive impairment (Jia et al., 2019; Janelidze et al., 2020).

Although there is no consensus on which individual miRNAs or groups of miRNAs from exosomes best predict cognitive impairment, some consistent evidence has emerged. For example, levels of miR-342-3p in plasma exosomes were significantly lower in AD participants compared to controls and correlated with poor cognitive performance (i.e., MoCA) among elderly, cognitively unimpaired individuals in a separate study (Lugli et al., 2015; Rani et al., 2017). Similarly, data across multiple cohorts indicate serum exosomes from AD individuals maintain lower levels of miR-193b (Liu et al., 2014; Yang et al., 2018). However, some inconsistent results have been reported; for instance, results suggest CSF exosomes from AD patients exhibit increased levels of miR-125b-5p, yet data from another study indicate its levels in plasma exosomes are associated with poor cognitive performance (Rani et al., 2017; McKeever et al., 2018). As there appears to be minimal concordance across studies, the application of exosomal miRNAs as reliable diagnostic indicators may currently be limited to within particular individuals (Dong et al., 2020).

In addition to cross-sectional investigations, several studies have used exosomes as biomarkers to predict longitudinal variation in cognitive decline. Using neurally derived exosomal concentrations of aggregate-prone peptides, researchers can predict individuals who progress from MCI to AD-dementia within 36 months (i.e., via MMSE scores), as well as cognitively sound individuals who develop dementia up to 10 years later (i.e., via CDR scores) (Fiandaca et al., 2015; Winston et al., 2016). Increased longitudinal accuracy can be achieved by combining such exosomal concentrations of aggregate-prone peptides with other performance measures (e.g., olfactory function) or other exosomal cargo (e.g., insulin receptor substrates; IRS) (Kapogiannis et al., 2015; Zhao et al., 2020). For instance, by coalescing measures of pTau and phosphorylated IRS-1 from exosome-enriched plasma samples, researchers can predict (i.e., >80% accuracy) elderly individuals who remain cognitively resilient from those who eventually display cognitive decline (i.e., mean follow up 3.5 ± 2.31 years) (Kapogiannis et al., 2019). It should be noted these aggregate-prone peptides in exosomes can also distinguish degrees of cognitive impairment in cross-sectional studies (i.e., healthy control vs. MCI vs. AD) (Jia et al., 2019; Nam et al., 2020; Perrotte et al., 2020). The most consistent results from neuronal-specific exosomes across different investigators and cohorts suggests a positive association between AD-specific biomarkers (i.e., Aβ, pTau) with cross-sectional and longitudinal risk of cognitive impairment (Fiandaca et al., 2015; Winston et al., 2016; Jia et al., 2019; Nam et al., 2020; Zhao et al., 2020).

To enhance the utilization of exosomes as reliable and valid biomarkers, several important limitations should be considered. When examining their associations with cognitive decline, researchers should take steps to differentiate the tissue-specific (i.e., systemic vs. brain) as well as the cell-specific origin of exosomes. Indeed, data indicate exosomes from the CNS contain significantly different molecular contents depending on an individual’s cognitive capacities (i.e., healthy controls vs. dementia) as well as their cellular origin (i.e., neurons vs. astrocytes), while such differences in content can subsequently induce distinct functional consequences for recipient cells (Goetzl et al., 2016b; Iguchi et al., 2016; Nogueras-Ortiz et al., 2020). Investigations should also consider steps to improve the diagnostic accuracy of exosomes for early stages of cognitive decline, given that current approaches differentiate healthy individuals from dementia cases more accurately than from MCI cases (Xing et al., 2021).

Despite limitations, the implementation of exosomes as biomarkers for age-related cognitive decline offers several advantages. As mentioned previously, exosomal sampling is more cost effective and less invasive than currently available biomarkers (i.e., CSF, PET). Furthermore, exosomes derived from the CSF and blood offer similar accuracy for identifying individuals exhibiting cognitive decline, while neuroimaging and blood derived exosomes exhibit similar specificity for distinguishing dementia cases from healthy controls (Gui et al., 2015; Jia et al., 2019; Lim et al., 2019). Meanwhile, exosomes can be sampled from several biological fluids (e.g., blood, saliva, urine, breast milk) (Pisitkun et al., 2004; Gallo et al., 2012; Mirza et al., 2019). Indeed, recent reports indicate neuronal exosomes can be isolated from saliva, while urinary exosomes can classify dementia patients from healthy controls (Rani et al., 2019; Sun et al., 2019). Additionally, the diversity of exosomal content presents investigators with the unique opportunity to improve diagnostic capacity; compared to approaches which assess a single molecular target (e.g., proteins), exosomes can enable researchers to leverage information simultaneously from a variety of molecular targets (e.g., proteins, lipids, RNA, DNA) (Thakur et al., 2014; Dinkins et al., 2017; Asada et al., 2019; Teruel-Montoya et al., 2019). However, comparisons across studies should be restricted to samples of the same biological fluid, given that preparations from different biological fluids incur differing degrees of contamination during preparation (Grigor’eva et al., 2017).

### Engineered Exosomes as a Cargo Delivery Vehicle for Cognition Therapeutics

Along with their diagnostic applications, exosomes can potentially serve as efficient delivery vehicles for the treatment of age-related cognitive dysfunction. Compared to traditional drug delivery, exosomes offer a number of benefits including their capacity to translocate functional biomolecules, their stability in blood, and immune tolerance (Pêche et al., 2006; Sun et al., 2013; Song et al., 2016). Naturally occurring exosomes derived from a variety of sources have been investigated for therapeutic applications, including those from different mammalian cells (e.g., mesenchymal stem cells, macrophages, tumor cells), plant cells, and biological fluids (e.g., plasma, serum, and milk) (Luan et al., 2017; Alfieri et al., 2021; Kučuk et al., 2021; Perut et al., 2021). Isolated exosomes can be exogenously loaded with therapeutic cargo for targeted delivery, ranging from RNAs (siRNA, miRNA, lncRNA) and proteins to synthetic chemicals and drugs. Numerous methodological approaches have been developed for modifying exosome contents for specific clinical applications, including passive (e.g., incubation) and active processes (e.g., sonication, extrusion, electroporation, chemical transfection) (Luan et al., 2017; Fu et al., 2020). Exosomes can also be modified to target a precise cell type within a given tissue by click chemistry methods that attach molecules of interest to the luminal surface via covalent bonds, or by manipulating exosome-producing parent cells *in vitro* (i.e., gene transfection, drug treatment) (Ohno et al., 2013; Smyth et al., 2014; Nakase and Futaki, 2015; Qi et al., 2016; Conlan et al., 2017). For instance, to generate exosomes that specifically target glioma cells, researchers have attached a neuropilin-1 peptide to cargo-loaded exosomes utilizing copper-catalyzed cycloaddition reactions of sulfonyl azide (Jia et al., 2018). Researchers have also developed transfection strategies to facilitate exosomal delivery to neurons and glia, such as fusing the rabies viral glycoprotein with the gene encoding for exosome transmembrane protein lysosome-associated membrane protein 2b (LAMP2B) (Alvarez-Erviti et al., 2011). Similar transfection strategies have enabled therapeutic delivery to glioma cells, whereby the fusion of a transferrin receptor peptide T7 with LAMP2B enabled the delivery of miRNA oligonucleotides against miR-21 (Kim et al., 2020).

Perhaps the greatest advantage offered by exosomes is their capacity to transverse the BBB due to their small size, endogenous biological properties, and low immunogenicity, which has otherwise been a major obstacle in treatment development for cognitive decline (Pardridge, 2020). This has enabled the systemic administration of exosomes to efficiently deliver genetic material (e.g., oligonucleotides, miRNAs) and therapeutic drugs to the brain, where they induce functional alterations in targeted CNS cell types (Alvarez-Erviti et al., 2011; Kim et al., 2020). Numerous animal studies have evaluated the potential use of exosomes as drug delivery mechanisms for age-related neurodegenerative disorders and neuroinflammatory conditions. Across several different mouse models, including those exposed to lipopolysaccharide (LPS)-induced CNS inflammation and experimental autoimmune encephalitis, the intranasal delivery and subsequent CNS uptake of exosomes loaded with anti-inflammatory compounds (i.e., curcumin or Stat3-inhibitor) protected mice from the deleterious effects otherwise associated with aberrant neuroinflammation (e.g., increased proinflammatory cytokine expression, increased disease severity) (Zhuang et al., 2011). Similarly, in a mouse model of ischemic stroke, the incorporation of neuron specific fusion proteins on the exosomal surface along with the intravenous application of such exosomes was capable of delivering specific compounds (e.g., miRNAs, flavonoids) to the CNS, where they exerted neuroprotective effects and promoted neuronal viability (Yang et al., 2017; Guo et al., 2021). Furthermore, evidence suggests exosomes can ferry cargo across the BBB without exogenous modification of CNS-targeting peptides on their surface. For instance, due to their inherent brain-homing peptides, exosomes isolated from macrophages and administered intravenously can deliver brain-derived neurotrophic factor (BDNF) to the CNS (Yuan et al., 2017). Likewise, systemic administration of macrophage-derived exosomes loaded with glial cell-line derived neurotrophic factor (GDNF) reduced neuroinflammation and ameliorated degeneration of dopaminergic neurons in a mouse model of Parkinson’s disease (PD) (Zhao et al., 2014). In another mouse model of PD, systemic administration of macrophage-derived exosomes delivered an antioxidant enzyme to dopaminergic neurons and resulted in significantly improved motor function (Haney et al., 2015). Beneficial effects have also been reported in transgenic AD-mice following intraparietal injection of brain-specific exosomes containing quercetin; here, exosomal delivery across the BBB resulted in an attenuation of memory and spatial learning deficits, as well as the mitigation of neuropathological hallmarks (e.g., pTau, neurofibrillary tangles) (Qi et al., 2020).

**FIGURE 1:**
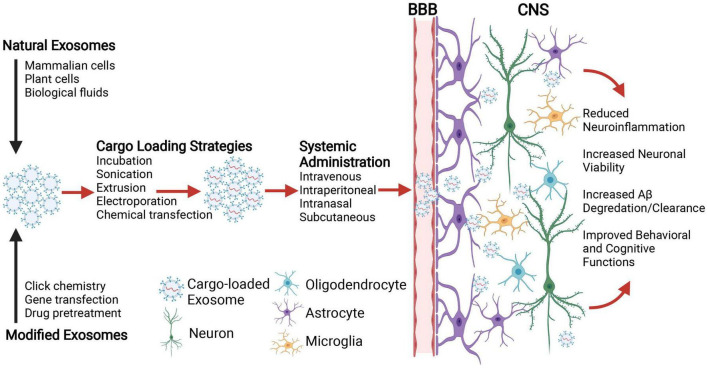
Schematic illustration depicting the application of exosomes for central nervous system (CNS) delivery of therapeutic cargo to combat the neurobiological underpinnings of age-related cognitive decline. Natural exosomes or those modified for targeted delivery to specific brain cells (e.g., neurons, oligodendrocytes, astrocytes, and microglia) are loaded with therapeutic molecular cargo (e.g., nucleic acids, proteins, and drugs) using passive or active strategies. Exosomes are then administered systemically, where the route of administration depends upon multiple parameters including stability, biodistribution, dose, and therapeutic efficacy. With their inherent capacity to penetrate the blood brain barrier (BBB), exosomes ferry therapeutic cargos to the targeted brain cells and ameliorate aberrant neurobiological processes (e.g., neuroinflammation, neuronal cell death, Aβ degradation/clearance etc.) that may otherwise contribute to age-related cognitive decline. Created with Biorender.

Although engineered exosomes may eventually prove more effective, existing evidence from stem cell derived exosomes demonstrates that even un-modified exosomes hold promise for dementia treatment. In APP/PS1 mice across several research groups, the application of exosomes isolated from cultured stem cells (human umbilical, mouse bone barrow, mouse embryonic) can reliably improve performance on cognitive tasks (novel object recognition, Morris Water Maze) and inhibit biological processes associated with cognitive impairment, including the dysregulated expression of pro- and anti-inflammatory cytokines (IL1-β and IL-10), the activation of CNS immune cells (astrocytes, microglia) and the deposition of Aβ (Aβ_1–40_, Aβ_1–42_, plaques) (Cui et al., 2018, 2019; Ding et al., 2018; Wang et al., 2018; Li et al., 2020; Yang et al., 2020). Additional data has suggested such protective effects observed across multiple AD mouse models may be due to the natural inclusion of antioxidants (bioactive catalase) and Aβ degrading enzymes (neprilysin, insulin degrading enzyme) in stem cell exosomes (de Godoy et al., 2018; Ding et al., 2018; Lee et al., 2018; Bodart-Santos et al., 2019; Elia et al., 2019; Micci et al., 2019; Apodaca et al., 2021). Together, these animal studies support the therapeutic potential of engineered exosomes as CNS-specific delivery vehicles to ameliorate cellular and cognitive dysfunction (Figure 1).

While the clinical development of therapeutic interventions faces a variety of hurdles, the consideration of exosomes as a novel delivery vehicle for future nanotherapeutics may prove to enhance outcomes for those individuals vulnerable to age-related cognitive pathologies. In this context, the use of un-modified exosomes isolated from adipose mesenchymal stem cells is currently being investigated for AD-dementia in clinics (NCT04388982). Although the study is only exploring the safety and efficacy of exosome treatment in patients, data from secondary outcome measures that include AD biomarkers based in Aβ plasma/CSF measures, PET scans, and neuropsychological tests, may further encourage the development of engineered vesicles for the treatment of age-related cognitive decline. Table 2 summarizes ongoing clinical trials using exosome-based therapeutic interventions in neurodegenerative and neurological disorders assessing cognitive, disability, and daily functioning outcomes^1^.

**TABLE 2:** Clinical trials involving exosome-based therapeutic interventions for neurological disorders (ClinicalTrials.gov Database, 2022).

Exosome Intervention	Route of administration	Therapeutic condition	Outcome measures	Clinical trial ID
Allogenic adipose MSC-derived exosomes	Intranasal	AD	Adverse event, cognitive function, quality of life, AD biomarkers	NCT04388982
Focused ultrasound delivery of exosomes	Intravenous	Refractory depression, anxiety disorders, and neurodegenerative dementia	Depressive and anxiety symptoms, cognitive function	NCT04202770
Allogenic MSC-derived exosomes transfected with miR-124	Stereotaxis/intraparenchymal	Cerebrovascular disorders	Stroke recurrence, seizure, hemorrhage, disability	NCT03384433
Exosomes containing neonatal stem cell products	Epineural using ultrasound guidance, intravenous	Neuralgia	Pain, depression severity, daily functioning, adverse event	NCT04202783

*AD, Alzheimer’s Disease; MSC, Mesenchymal Stem Cells.*

## Conclusion

The current composition proposes exosomes are uniquely suited to improve our mechanistic understanding as well as enhance patient outcomes for the growing public health challenge of age-related cognitive decline. We highlight how the analysis of exosomes can improve our understanding of varying mechanisms in age-related cognitive decline, including the role of miRNAs, neuroinflammation and aggregate-prone proteins (i.e., Aβ, pTau). Despite these contributions, we suggest specific areas of inquiry that warrant further interrogation, including the elucidation mechanisms in recipient cells that are modulated by exosomal miRNAs, the characterization of exosomal contents that are responsible for alterations in neuroinflammation and the examination of other aggregate-prone peptides (e.g., TDP-43, α-synuclein) in exosomes that may synergistically increase risk for late life cognitive impairment. Moreover, we discuss how exosomes might be used to enhance outcomes for elderly individuals by serving as reliable biomarkers and therapeutic agents.

To improve the reliability of studies going forward, consistent nomenclature coupled with protocol modifications that enhance the discrimination of EVs could be particularly effective. Due to similar biophysical properties (i.e., overlapping size, density, and protein compositions), isolation techniques developed for exosomes can inadvertently lead to results that are derived from samples containing a variety of different EVs (Théry et al., 2018; Witwer and Théry, 2019). Similarly, exosome-specific effects can be reported from protocols that fail to adequately purify EVs from non-vesicular components that can confound ensuing biochemical measurements (e.g., RNA-protein complexes) (Lötvall et al., 2014). To avoid different kinds of vesicles being labeled with the same terminology, MISEV guidelines suggest EVs should instead be labeled according to their size, biochemical composition and conditions/cellular origin (Théry et al., 2018). Several methodological alterations have also been suggested to improve accuracy in terminology, including the use of serum over plasma, and the use of brain derived exosomes rather than total exosomes (Xing et al., 2021). Moreover, technical aspects should also be considered for increasing reliability, including the standardization of exosomal extraction and isolation, where differences in protocols can otherwise lead to substantially divergent results both between as well as within procedures (e.g., immunoprecipitation, ultracentrifugation) (Martins et al., 2018; Patel et al., 2019; Brennan et al., 2020; Shtam et al., 2020). Similarly, investigations should employ parallel protocols for the handling and preservation of samples across experimental designs, given that alternative storage conditions can alter exosome stability (Cheng et al., 2019). Consistent nomenclature suggested by MISEV guidelines, protocol adjustments (i.e., using appropriate controls, characterizing vesicles with advanced microscopy, profiling protein composition etc.) and standardized technical procedures across studies may improve the reliability of exosome measurements and enhance their application in the context of age-related cognitive decline.

Several additional limitations hinder the further application of exosomes discussed herein. Notably, it remains unknown whether concurrent variation in exosomes and cognitive capacities is reflective of a causal or reverse-causal association. In other words, it is difficult to determine if exosomal differentiation contributes to divergent cognitive trajectories, or if such differences are secondary to other biological processes that otherwise increase risk for cognitive deterioration in aging. A lack of data measuring time-dependent variation in the profiles of neuronal exosomes contributes to this lack of understanding, and potentially inhibits researchers from adequately controlling for the effects of aging. Although a limited set of investigations have employed longitudinal designs (Fiandaca et al., 2015; Winston et al., 2016; Kapogiannis et al., 2019; Zhao et al., 2020), future studies should take steps to establish temporal precedence and determine the causal implications of exosomes in facilitating cognitive decline. Along with the call to establish causation, additional evidence is needed to discern the role of modifiable life factors that can lead to cognitive resiliency in aging, such as exercise, diet and cognitive stimulation (Duggan and Parikh, 2021). For example, our recent data in rodents has demonstrated that engagement on an attention demanding task during aging leads to the upregulation of transcripts linked to extracellular vesicles, suggesting exosome-related biochemical pathways could be important for inducing individual variation in cognitive resilience (Duggan et al., 2019). Another limitation is the need to examine alternative neurobiological mechanisms not discussed in the current composition. Indeed, data suggest individuals exhibiting cognitive deficits in aging maintain altered concentrations of exosomal proteins implicated in insulin signaling (Kapogiannis et al., 2015, 2019), lysosomal degradation/autophagy (Goetzl et al., 2015) as well as synaptic integrity (Goetzl et al., 2016a,2018a; Winston et al., 2016, 2018; Agliardi et al., 2019; Jia et al., 2021). By addressing these limitations, researchers may gain a greater understanding of exosome-mediated regulation of miRNAs, neuroinflammation, and aggregate-prone proteins, while enhancing the potential for exosomes to serve as reliable biomarkers of cognitive decline and as nanocarriers to deliver therapeutic agents to the brain in neurodegenerative conditions.

## Author Contributions

MD and VP conceived and designed the study. MD and AL conducted the literature search. MD, AL, and VP wrote the manuscript. TF and MW edited the final manuscript. All authors have read and approved the manuscript.

## Conflict of Interest

The authors declare that the research was conducted in the absence of any commercial or financial relationships that could be construed as a potential conflict of interest.

## Publisher’s Note

All claims expressed in this article are solely those of the authors and do not necessarily represent those of their affiliated organizations, or those of the publisher, the editors and the reviewers. Any product that may be evaluated in this article, or claim that may be made by its manufacturer, is not guaranteed or endorsed by the publisher.

## Footnotes

1
http://www.clinicaltrials.gov
